# Fundamental Study on the Development of Structural Lightweight Concrete by Using Normal Coarse Aggregate and Foaming Agent

**DOI:** 10.3390/ma7064536

**Published:** 2014-06-13

**Authors:** Han-Seung Lee, Mohamed A. Ismail, Young-Je Woo, Tae-Beom Min, Hyun-Kook Choi

**Affiliations:** 1 Department of Architectural Engineering, Hanyang University, 1271 Sa 3-dong, Sangrok-gu, Ansan 426-791, Korea; E-Mails: ercleehs@hanyang.ac.kr (H.-S.L.); imgod@kcl.re.kr (Y.-J.W.); nsu02@hanyang.ac.kr (T.-B.M.); 2 R & D Center, Sungshin Cement, 48-37 Bugang Oecheon-ro, Bugang-Myun 339-940, Korea; E-Mail: hkchoi@sscem.com

**Keywords:** microporous materials, mechanical testing, mechanical properties, elastic properties, microstructure

## Abstract

Structural lightweight concrete (SLWC) has superior properties that allow the optimization of super tall structure systems for the process of design. Because of the limited supply of lightweight aggregates in Korea, the development of structural lightweight concrete without lightweight aggregates is needed. The physical and mechanical properties of specimens that were cast using normal coarse aggregates and different mixing ratios of foaming agent to evaluate the possibility of creating structural lightweight concrete were investigated. The results show that the density of SLWC decreases as the dosage of foaming agent increases up to a dosage of 0.6%, as observed by SEM. It was also observed that the foaming agent induced well separated pores, and that the size of the pores ranged from 50 to 100 μm. Based on the porosity of concrete specimens with foaming agent, compressive strength values of structural lightweight foam concrete (SLWFC) were obtained. It was also found that the estimated values from proposed equations for compressive strength and modulus of elasticity of SLWFC, and values obtained by actual measurements were in good agreement. Thus, this study confirms that new structural lightweight concrete using normal coarse aggregates and foaming agent can be developed successfully.

## 1. Introduction

Compared to normal and heavyweight concrete, structural lightweight concrete (SLWC) has the advantage of having a finer density [1]. Therefore, the application of SLWC in skyscrapers of residential and commercial complexes, as well as office buildings would reduce the overall weight of the buildings, reduce the amount of material used and reduce seismic and wind loads. This is expected to have an effect on the optimization of the structural systems of skyscrapers in terms of energy efficiency and materials savings [2]. Generally, lightweight aggregate is used for the manufacturing of lightweight concrete, but in Korea, it is extremely difficult to have mass manufacturing of lightweight aggregates, predominatly because of the limited number of available manufacturers. In addition, lightweight concrete is only used for non-structural purposes [3]. Therefore, development of SLWC by using a foaming agent mixed with normal coarse aggregates rather than lightweight aggregates would enable the use of massive lightweight concrete for different structural applications. 

**Figure 1:**
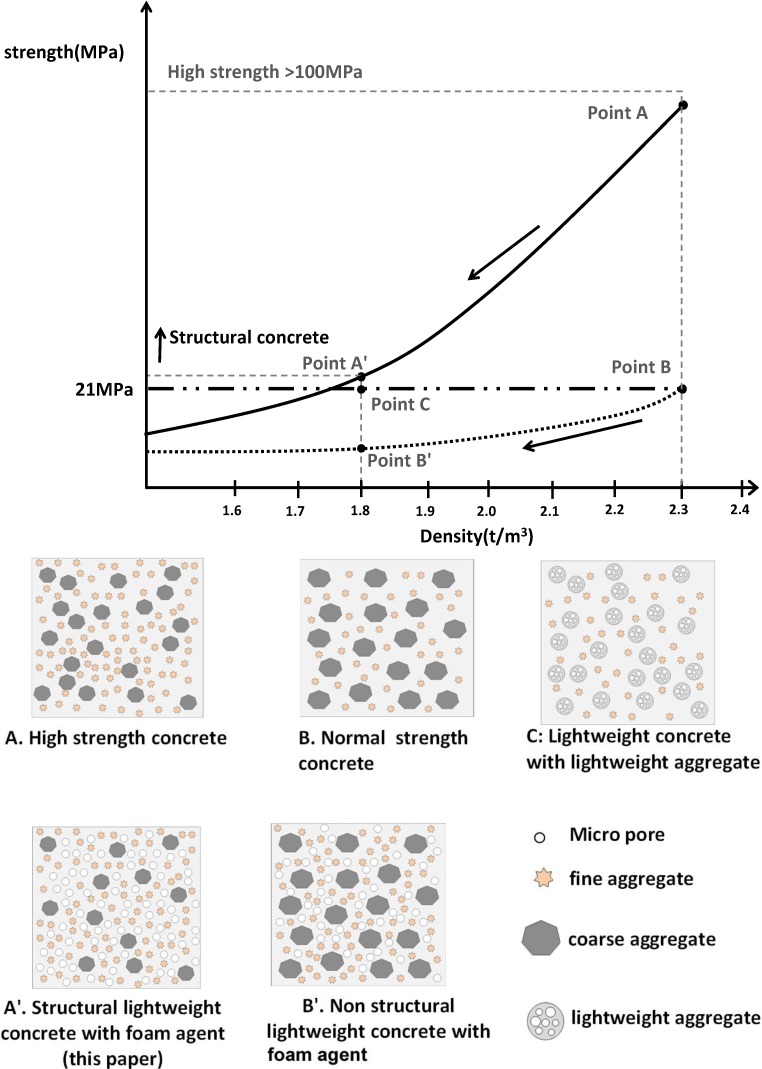
Concept of structural lightweight foamed concrete used in this study.

Figure 1 shows the basic concept of this study. Point A refers to high strength concrete of more than 100 MPa at a density of 2.3 t/m^3^. This compressive strength is usually obtained by adding silica fume and/or fly ash to the concrete mix besides using normal coarse and fine aggregates and superplasticizers. Point B refers to normal strength concrete and point C refers to lightweight concrete that is produced from lightweight aggregates. What is proposed in this study is that structural lightweight concrete can be produced by using coarse aggregates, whereas weight restrictions can be resolved by introducing a foaming agent to the high strength concrete mix. Point A' refers to SLWC with foaming agent and point B' is the nonstructural lightweight concrete with foaming agent. In Korea, the minimal compressive strength that can be used in structural elements is 21 MPa, and it is proposed in this study to develop structural lightweight foamed concrete with a compressive strength that is higher than the compressive strength of reinforced concrete slabs.

To this end, specimens of mortar with foaming agent (hereinafter denoted structural lightweight foamed mortar: SLWFM) were constructed to evaluate their physical and mechanical characteristics. Based on the test outcome, specimens of structural lightweight foamed concrete using coarse aggregates (hereinafter denoted structural lightweight foamed concrete: SLWFC) were developed in accordance with the SLWFM physical characteristics. The compressive strength correlation function of lightweight foamed concrete casts with different foaming agent dosage was evaluated to obtain basic data for the development of SLWFC [4,5,6].

## 2. Tests on Structural Lightweight Foamed Mortar

### 2.1. Test Overview

In these experiments, the compressive strength of mortar without foaming agent was set as high compressive strength. The ratios of W/B were 20%, 25% and 30%. The added dosages of foaming agent were set at 0%, 0.3%, 0.6% and 0.9% of binder weight. Also, for the sake of comparison, general mortar specimens of W/C = 60% were prepared [7,8].

Table 1 shows the physical properties of ordinary Portland cement and silica fume used in this study. Table 2 shows the chemical composition of ordinary Portland cement, silica fume and blast furnace slag (BFS) used in this study. Table 3 shows the physical properties of coarse and fine aggregates. The percentage of solids and fineness modulus of fine aggregates are 62.6% and 2.81, respectively.

Table 4 shows the physical properties of the plasticizers and the foaming agent used. Table 5 shows the mix design of SLWFM.

Mortar specimens of dimension 40 mm × 40 mm × 160 mm were prepared according to KS L ISO 679 [9] and were tested in triplicate for compressive strength after 7 and 28 days.

**Table 1:** Physical properties of ordinary Portland cement and silica fume.

Material/test type	Ordinary Portland cement	Silica fume
Specific gravity	3.05	2.2
Fineness (cm^2^/g)	3,357	–
BET surface area	–	200,000
Initial setting (min)	110	–

**Table 2:** Chemical composition of ordinary Portland cement, silica fume and BFS.

Component	Ordinary Portland cement (%)	Silica fume (%)	BFS, wt%
SiO_2_	21.94	93.2	34.5
Al_2_O_3_	4.95	0.44	13.74
Fe_2_O_3_	3.74	0.20	0.97
CaO	62.33	–	42.1
MgO	2.08	0.81	7.29
SO_3_	2.22	0.45	–
K_2_O	0.56	1.28	0.49
Na_2_O	0.32	0.59	0.22
TiO_2_	0.17	–	–
Mn_2_O_3_	0.05	–	–
CI^−^	0.01	–	0.022
LOI	1.78	–	−1.05
IR	0.24	–	–
C	–	0.55	–
C(isolated)	–	0.41	–
pH	–	7.9	–
Si(isolated)	–	0.29	–
SiC	–	0.50	–

**Table 3:** Physical properties of coarse and fine aggregates.

Type	Aggregate size	Absorption (%)	Density (g/cm^3^)	Unit weight (kg/m^3^)
Coarse aggregates	25 mm	1.79	2.60	1530
Fine aggregates	<5 mm	2.43	2.55	1677

**Table 4:** Physical properties of superplasticizer and foaming agent.

Main component	Type	Density (g/cm^2^)	Usage (wt% of Binder)	Viscosity			
				Spindle (NO)	Torque (N·m)	RPM
PC (polycarboxilic)	Liquid	1.05	1~4	1	934	140
Silica composite mineral		1.03~1.04	0.3~0.9	–	–	–

**Table 5:** Structural lightweight foamed mortar (SLWFM) mix design.

No.	W/B (%)	Fa (%)	Fa (kg/m^3^)	Weight mixing (kg/m^3^)				AD %
				W	C	SF	S	
20-0	20	0	0	150	603	139	452	1.9
20-0.3		0.3	2.22					
20-0.6		0.6	4.45					
20-0.9		0.9	6.67					
25-0	25	0	0	170	575	95	452	1.4
25-0.3		0.3	2.01					
25-0.6		0.6	4.02					
25-0.9		0.9	6.03					
30-0	30	0	0	180	520	75	452	0.9
30-0.3		0.3	1.78					
30-0.6		0.6	3.57					
30-0.9		0.9	5.35					
60	60	–	–	156	260	–	86	–

### 2.2. Test Method and Results of Structural Lightweight Foamed Mortar

**Figure 2:**
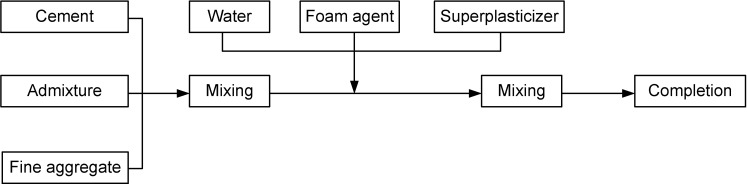
Manufacturing method for structural lightweight foamed mortar (SLWFM) applied in this study.

Mortar specimens were cast in the study according to KS L ISO 679 [9] (hydraulic cement mortar compressive strength test method). Among manufacturing methods for lightweight foamed concrete, the post-foaming method, which is deemed as highly feasible for concrete manufacturing, was adopted and the order of procedures is as shown in Figure 2. As for curing, water curing was conducted until the test age [4].

#### 2.2.1. The Dosage of Foam Agent and Density

The apparent density for structural lightweight foamed mortar was calculated according to KS F 4039 [10] as follows:

Structural lightweight foamed mortar density = W_s_/1000 (1)

where W_s_ is specimen weight in 1000 mL.

**Figure 3:**
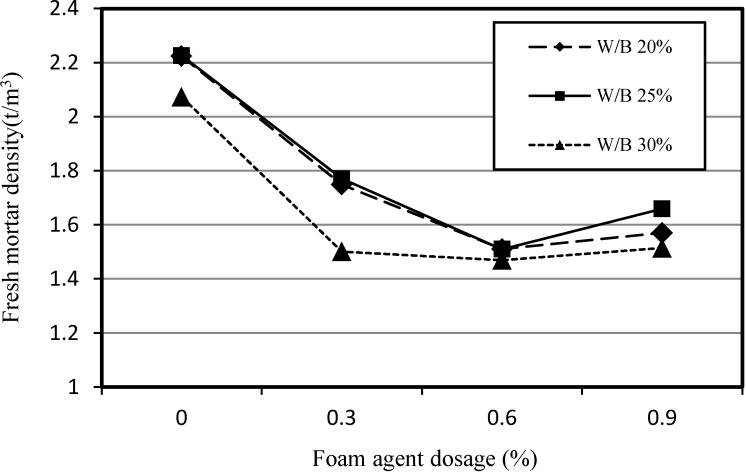
Relationship between apparent density of fresh SLWFM and foaming agent dosage.

Figure 3 shows the relationship between the density of fresh SLWFM and the dosage of the foaming agent for W/B ratios of 20%, 25% and 30%.

It was observed that the increase in the dosage of the foaming agent resulted in a decrease in the density of fresh SLWFM up to 0.6%. Subsequently, an increase at a dosage of 0.9% was observed. Furthermore, above a dosage of 0.60%, an increase in the foaming agent dosage did not substantiality cause any deterioration of the density due to the foam and/or foam fraction. Therefore, a foaming agent dosage of 0.6% was selected as the maximum foaming agent dosage value that can be used. Similar results were obtained with W/B ratios of 20%, 25% and 30%.

#### 2.2.2. Apparent Density of Structural Lightweight Foamed Mortar

The specimen’s apparent density under dry surface conditions was calculated according to Equations 1 and 2, and the average of three specimens was determined.

Mortar apparent density in surface dry condition = *w*_0_/*V*·*p*_w_ (2)

where *p*_w_ is the density of water (g/cm^3^)

**Figure 4:**
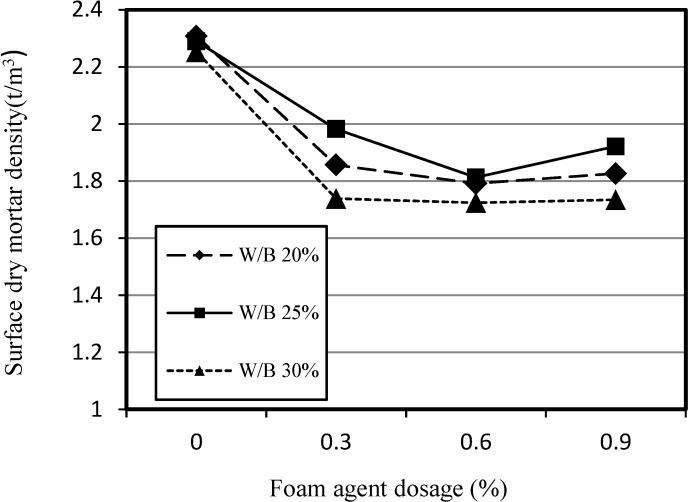
Relationship between dry surface density of SLWFM and foaming agent dosage.

Figure 4 shows the relationship between the apparent density of SLWFM and the dosage of the foaming agent for W/B ratios of 20%, 25% and 30% under dry surface conditions. The increase in the dosage of foaming agent resulted in a decrease in the apparent density up to 0.6%, regardless of the W/B ratio. However, above 0.6%, a tendency of the apparent density to increase due to linkage with the foaming agent fraction during mortar mixing was observed.

This phenomenon became more pronounced with higher W/B ratio. In order to prevent the fraction or linking of the foam agent, either the W/B ratio must be decreased the viscosity of mortar must be increased. The dry surface density of specimens shows a high apparent density, because of water accumulation in the internal foam, and consequently it is recommended to apply the product in construction sites under sustained dry conditions.

#### 2.2.3. Porosity of Structural Lightweight Foamed Mortar

**Figure 5:**
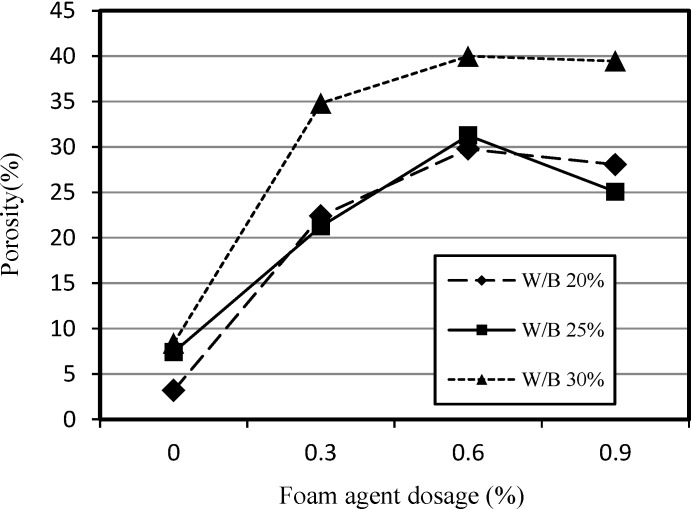
Relationship between porosity of SLWFM and foaming agent dosage.

Figure 5 shows the relationship between the porosity and the dosage of foaming agent. The increase in the dosage of foaming agent resulted in an increase in porosity up to 0.6% of foaming agent dosage. For W/B ratios of 20% and 25%, there was a moderate increase in the porosity in comparison with a W/B ratio of 30% up to a foaming agent dosage of 0.6%. At a foaming agent dosage of 0.9%, the porosity started to decrease moderately for W/B ratios of 20% and 25%, and slightly for a W/B ratio of 30%. The later may be due to the friction between sand and water when relatively large amounts of water are used.

**Figure 6:**
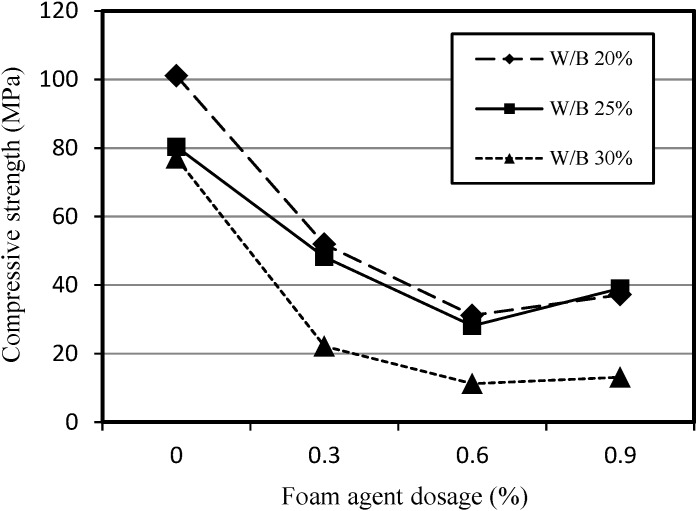
Relationship between compressive strength of SLWFM and foaming agent dosage.

Figure 6 shows the relationship between the compressive strength of SLWFM and the dosage of foaming agent used. It is clear that as the percentage of foaming agent increases, the compressive strength decreases due to the fact that the higher percentage of foaming agent induces an increase in the porosity, and subsequently, this results in the deterioration of compressive strength. At a foaming agent dosage of 0.9%, the pores become interconnected, and as a result, the pore holes decrease and the matrix of the mortar increases in strength due to the reduction in separate pores.

**Figure 7:**
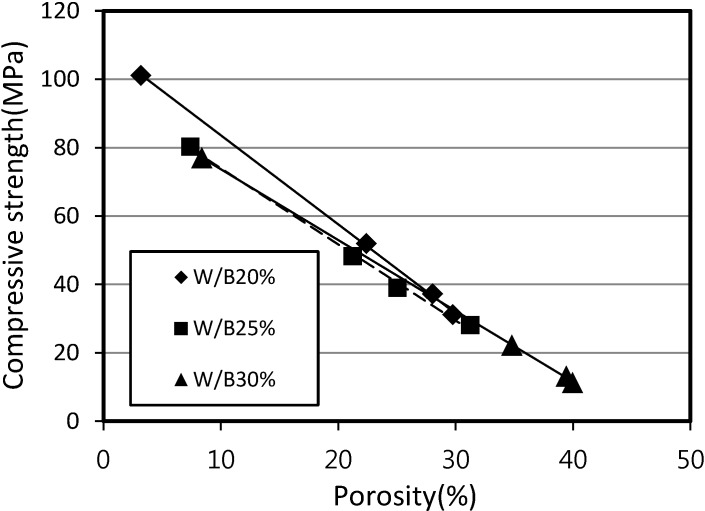
Relationship between compressive strength of SLWFM and porosity at different W/B ratios.

Figure 7 shows the relationship between the compressive strength and the porosity of SLWFM. It is clear that regardless of W/B ratio, the increase in porosity within mortars causes the compressive strength to linearly decrease. Table 6 shows the physical and mechanical properties of hardened SLWFM.

**Table 6:** Physical and mechanical properties of hardened SLWFM.

Description	Compressive strength (MPa)		Apparent density (t/m^3^)			Porosity (%)
	7 days	28 days	Fresh	surface dry	overdry condition	
20-0	74.9	101.05	2.223	2.307	2.233	3.2
20-0.3	32.37	51.88	1.748	1.856	1.780	22.4
20-0.6	27.67	31.17	1.510	1.791	1.620	29.77
20-0.9	30.03	37.18	1.570	1.826	1.660	28.04
25-0	72.93	80.32	2.226	2.289	2.236	7.41
25-0.3	39.33	48.21	1.770	1.982	1.817	21.24
25-0.6	22	28.07	1.509	1.812	1.586	31.25
25-0.9	34.7	39.00	1.659	1.921	1.729	25.05
30-0	54.63	77.01	2.073	2.252	2.157	8.4
30-0.3	16.33	22.19	1.501	1.738	1.604	34.81
30-0.6	9.3	11.22	1.469	1.724	1.385	39.97
30-0.9	11.80	13.12	1.514	1.734	1.497	39.45
60	9.42	21.46	2.064	2.112	1.902	9.94

Notes: 20 = W/B 20%; 25 = W/B 25%; 30 = W/B 30%.

## 3. Structural Lightweight Foamed Concrete Tests

Based on existing studies on SLWFM, the following targets were set in this study to define the applicable mixing scope for structural purposes: (i) to determine the basic characteristics; (ii) to evaluate the relevant mixing based on the characteristics in order to assess the application feasibility; and (iii) to develop an estimated correlation function between compressive strength test outcomes of SLWFM and compressive strength of SLWFC. 

**Figure 8:**
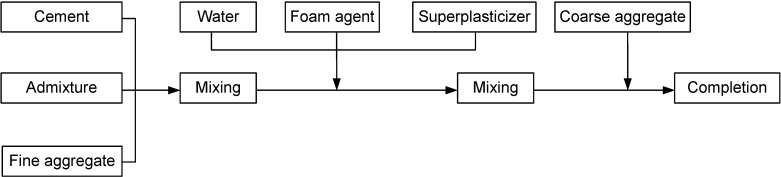
Manufacturing method for structural lightweight foamed concrete applied in this study.

In this study, the concrete test objectives were developed according to KS F 2403 [11]. Among manufacturing methods for SLWFC, the post-foam method was adopted, and the order in which the mixing was done is shown is shown in Figure 8. As for curing, water curing was conducted up to the test age. Table 7 shows the mix design of SLWFC. Concrete cylinder specimens of dimension 100 mm × 200 mm were prepared according to KS F 2403 [11] and were tested for compressive strength in triplicate after 3, 7 and 28 days. Table 8 shows the physical and mechanical characteristics of hardened SLWFC.

**Table 7:** Mix design of SLWFC.

No.	W/B (%)	Fa (%)	Fa (kg/m^3^)	Weight mixing (kg/m^3^)						
				W (kg/m^3^)	C (kg/m^3^)	Silica Fume (kg/m^3^)	BFS (kg/m^3^)	S (kg/m^3^)	G (kg/m^3^)	AD (%)
17-0	17	0	0	155	603	139	186	452	880	2.5
17-0.1		0.1	0.7							
17-0.3		0.3	2.2							
17-0.6		0.6	4.45							
17-0.9		0.9	6.67							
24-0	24	0	0	168	503	85.1	108	759	1101	1
24-0.3		0.3	1.76							
24-0.6		0.6	3.52							
60-0	60	0	0	156	260	0	0	867	1004	0.5

**Table 8:** Physical and mechanical properties of hardened SLWFC.

Description	Compressive strength (MPa)			Apparent density (t/m^3^)			Porosity (%)
	3 days	7 days	28 days	Fresh	Surface dry	Overdry condition	
17-0	68.8	76.5	91.3	2.42	2.48	2.36	4.84
17-0.1	53.65	55.9	70.58	2.14	2.2	2.14	13.71
17-0.3	36.30	36.3	60.32	1.95	2.08	2.0	19.35
17-0.6	24.60	36	43.15	1.78	1.95	1.87	24.60
17-0.9	29.03	15.6	19.5	1.70	1.79	1.76	29.03
24-0	54.70	60.0	77.55	2.35	2.31	2.28	1.30
24-0.3	43.40	46.2	59.25	1.88	2.14	2.1	9.09
24-0.6	20.90	22.7	30.1	1.67	1.96	1.9	17.75
60-0	17.55	20.15	30.25	2.41	2.4	2.31	3.71
60-0.3	8.75	9.65	14.80	1.92	2.1	1.91	20.42
60-0.6	8.05	9.45	13.40	1.71	1.94	1.85	22.92

### 3.1. Dosage of Foaming Agent and Density

The apparent density measurement of SLWFC was conducted in line with KS F 2409 [12] and was calculated as follows:

Structural lightweight foamed concrete density (*M*) = *W*/*V* (3)

where *M* is the unit volume mass of concrete (kg/m^3^)

**Figure 9:**
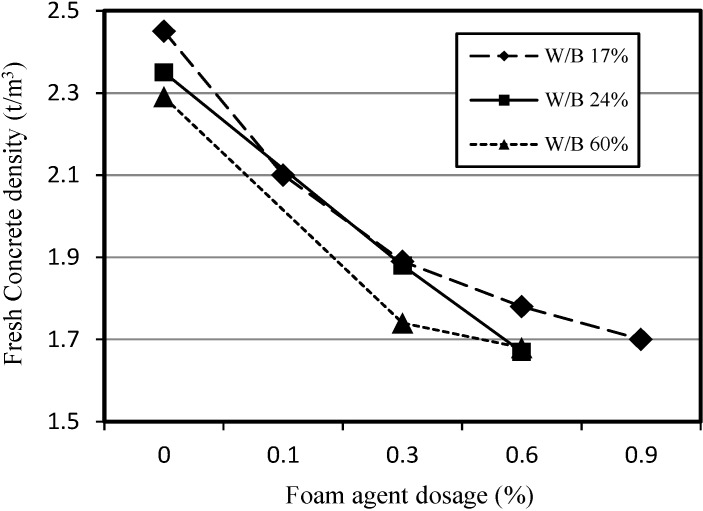
Relationship between apparent density of fresh SLWFC and foaming agent dosage.

Figure 9 shows the relationship between the apparent density of fresh SLWFC and the dosage of foaming agent. The density of fresh SLWFC was affected by the dosage of the foaming agent for all W/B ratios. The increase of the foaming agent dosage caused a decrease in the apparent density, and this occured for all W/B ratios. Because it was observed that the density decreased with increasing foaming agent dosage, which was in agreement with existing studies [13,14], a foaming agent dosage of 0.6% as selected as the maximum foaming agent dosage for use in SLWFC in order to secure the maximum porosity *versus* compressive strength.

### 3.2. Porosity and Compressive Strength Dependency on Density of Structural Lightweight Foamed Concrete

**Figure 10:**
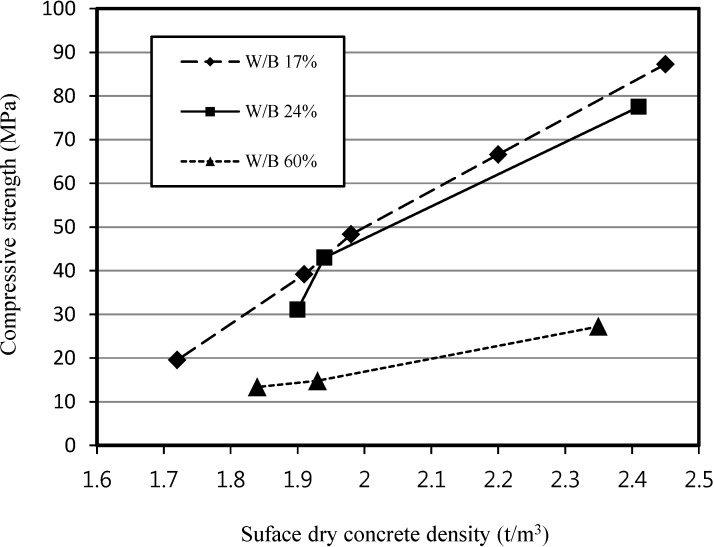
Relationship between compressive strength and surface dry concrete density of SLWFC.

Figure 10 shows the relationship between compressive strength and surface dry density at different W/B ratios. It is clear that as the density increased, the compressive strength increased for W/B ratios of 17%, 24% and 60%.

Test outcomes on compressive strength showed that the dosage of the foaming agent in the range between 0.3% and 0.6% for W/B 17% and W/B 24% was appropriate to manufacture LWFC for structural purposes.

Similar to the observed reduction in the density in relation to the increase in foaming agent dosage for SLWFM in previous studies, the density reduction in SLWFC concrete was found to follow similar patterns.

**Figure 11:**
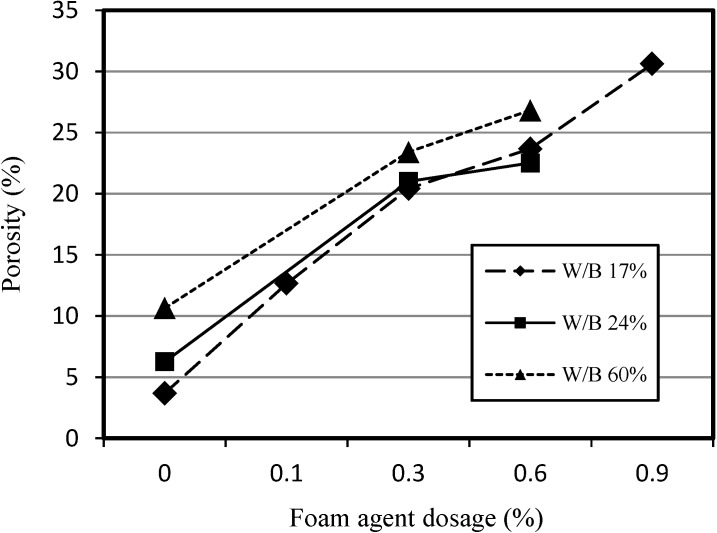
Relationship between porosity and foaming agent dosage for SLWFC.

Figure 11 shows the relationship between porosity and the dosage of foaming agent for SLWFC at different W/B ratios. As the dosage of the foaming agent increased, the porosity increased. It is, however, noticed that the porosity difference was not large due to foam sustainability and fractures. In addition, higher W/B ratio at the same foaming agent dosage resulted in higher porosity levels, since foams are caused by friction of sand and water under the condition of relatively large amounts of water.

**Figure 12:**
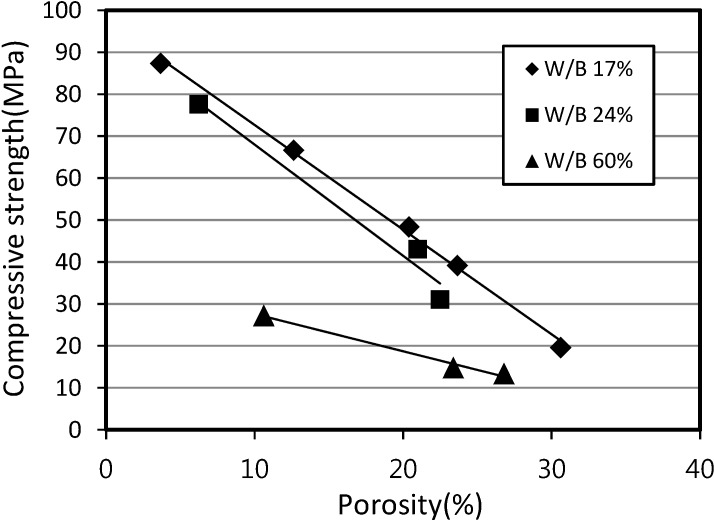
Relationship between compressive strength and porosity for SLWFC.

Figure 12 shows the relationship between the compressive strength and the porosity of SLWFC. The results illustrate the fact that the compressive strength deteriorated linearly as porosity increased. The reduction in values of SLWFM in existing tests was similar in pattern to those of SLWFC. Therefore, it may be assumed that the compressive strength can be controlled through the porosity, and the strength (FC) estimation of SLWFC at 0% porosity can be estimated from the trend line.

### 3.3. Modulus of Elasticity Measurement of Structural Lightweight Foamed Concrete

**Figure 13:**
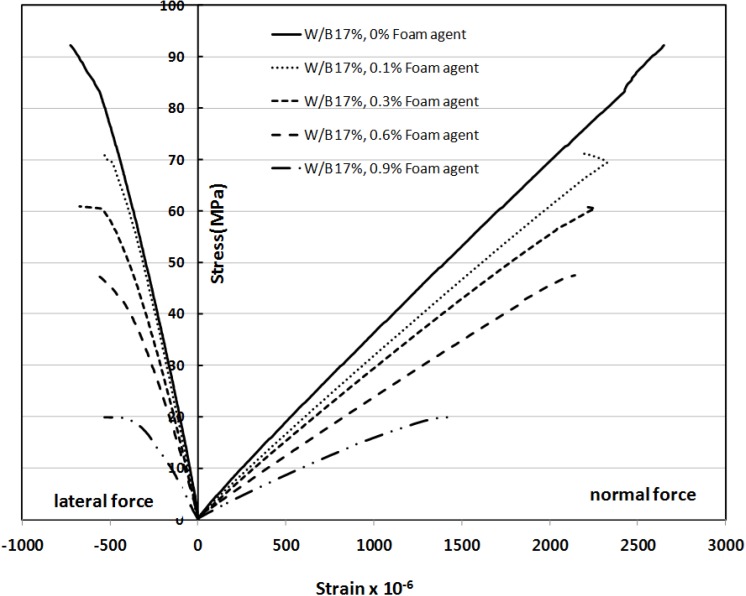
Relationship between stress and strain of SLWFC at W/B 17% and different foaming agent dosage.

Figure 13 shows the relationship between stress and strain for SLWFC at a W/B ratio of 17% and with different foaming agent dosages. With increasing stress, the measured strain increased. It is also apparent that at the same stress value, the strain value increased as the foaming agent dosage increased, and thus the modulus of elasticity decreased. It is, however, noticeable that up to a foam dosage of 0.60%, the change in strain values is relatively small, whilst it is largely increases at a value of 0.9%. It is also found that the structural lightweight concrete’s elastic modulus trend shows a similar trend as for normal concrete.

Because the increase in the foaming agent dosage results in a decrease in the stress, the modulus of elasticity decreases as well. It was found that the measured values of modulus of elasticity and compressive strength of SLWFC from this study matched the values calculated using Equations 4 and 5 suggested in concrete structural design standard explanation book [15].

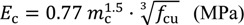
(4)

*f*_cu_ = *f*_ck_ + 8 (MPa) (5)

where *f*_ck_ is concrete compressive strength; *m*_c_ is concrete density in kg/m^3^.

Table 9 shows the comparison of the elasticity modulus for estimated values from Equations 4 and 5 and tested values. Both values are in close agreement. It is also clear that as the W/B ratio increases, the compressive strength values decrease, and thus the modulus of elasticity decreases. Also, for the same W/B ratio and under different foaming agent dosages, the higher the dosage, the lower the compressive strength and thus the modulus of elasticity.

Table 10 shows the density and porosity values of hardened SLWC. The porosity increases as the W/B ratio increases, whilst the density decreases. Also, at the same W/B ratio, the higher the foaming agent dosage, the higher the porosity and the lower the density.

**Table 9:** Comparison of tested modulus of elasticity of SLWFC and estimated values.

Description	Compressive strength (MPa)	Tested modulus of elasticity (GPa)	Structure design criteria estimated modulus of elasticity (GPa)
17-0	87.3	3.66	3.92
17-0.1	66.58	3.06	3.34
17-0.3	48.32	2.77	2.60
17-0.6	39.15	2.23	2.33
17-0.9	19.55	1.61	1.65
24-0	77.55	3.34	3.74
24-0.3	43	2.57	2.43
24-0.6	31.1	1.89	2.16
60-0	27.25	2.66	1.71
60-0.3	14.8	1.46	1.70
60-0.6	13.4	1.34	1.68

**Table 10:** Density and porosity of hardened SLWFC.

Description	Apparent density (t/m^3^)			Porosity
	Fresh density	Surface dry density	Over dry condition density	
17-0	2.45	2.45	2.36	3.67
17-0.1	2.1	2.2	2.14	12.65
17-0.3	1.89	1.98	1.95	20.41
17-0.6	1.78	1.91	1.87	23.67
17-0.9	1.70	1.72	1.7	30.61
24-0	2.35	2.41	2.28	6.25
24-0.3	1.88	1.94	1.92	20.99
24-0.6	1.67	1.9	1.86	25.1
60-0	2.29	2.35	2.12	10.64
60-0.3	1.74	1.83	1.8	23.40
60-0.6	1.68	1.84	1.72	26.81

### 3.4. Size and Distribution of Foaming Agent and Fine Pores

**Figure 14:**
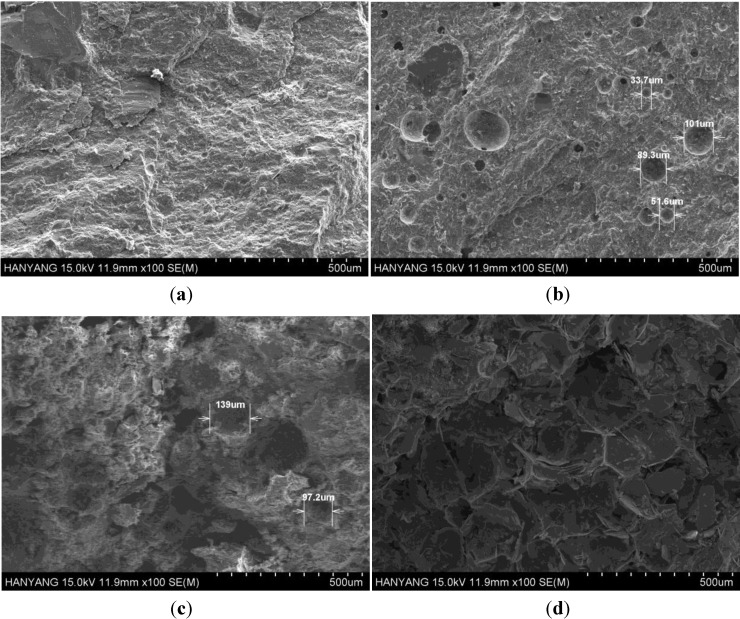
SEM images of fine pore structure of structural lightweight foamed concrete (W/B 17%) at foaming dosages of (**a**) 0%; (**b**) 0.3%; (**c**) 0.6%; (**d**) 0.9%.

Figure 14 shows SEM images magnified 100 times of the pore distribution changes and size of the pores in parts of hardened concrete of W/B 17% with different foaming agent dosages. It is clear that such observations cannot confirm the existence of isolated pores in W/B 17% specimen without foaming agent, but in specimens with foaming agent, isolated pores due to the presence of the foaming agent are evident, as seen from Figure 14b–c. At a dosage of 0.9%, the size of the pores and the intensity of the pores increase to such an extent that interconnected pores are formed (Figure 14d). Therefore, based on the results from SEM, the proper amount of foaming agent was deemed to be 0.6% of binder weight.

## 4. Compressive Strength Estimation for Structural Lightweight Foamed Mortar and Structural Lightweight Foamed Concrete

**Figure 15:**
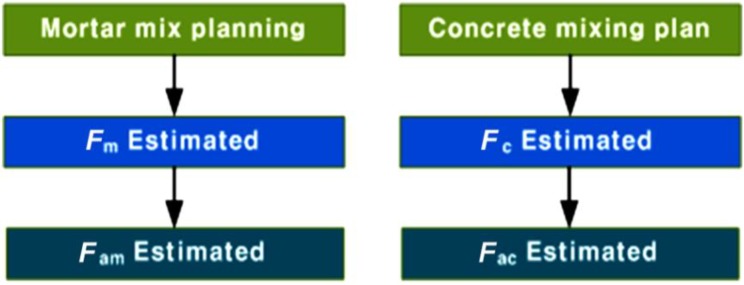
Estimation steps of *F*_am_ and *F*_ac_ from *F*_m_ and *F*_c_.

Figure 15 shows the steps used in the estimation of the compressive strength of SLWFM and SLWFC to determine the porosity and compressive strength correlation function.

After the completion of casts of mortar and concrete specimens, compression tests were conducted, and the values of compressive strength of mortar and concrete without foaming agent (*F*_m_ and *F*_c_) were then calculated. The porosity (*A*_p_) dependence on the foaming agent was also calculated in order to estimate the compressive strength of foamed agent mortar (*F*_am_) and foamed agent concrete (*F*_ac_) of SLWFM and SLWFC.

### 4.1. Estimation of F_am_ and F_ac_

The porosity determination of mortar and concrete with foaming agent is necessary to estimate the compressive strength of mortar and concrete with foaming agent. From Figure 7 and Figure 12, the values of the compressive strength of mortar and concrete can be calculated from the regression trend line formula *y* at a porosity value of 0%. This can be done by substituting *x* with 0. The compressive strength values of three trend lines at W/B of 20%, 25% and 30% were obtained.

**Figure 16:**
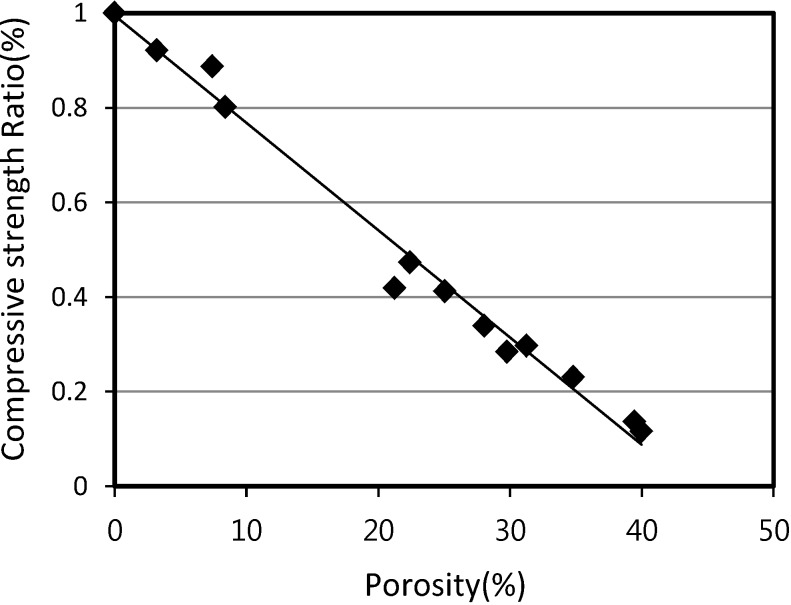
Relationship between compressive strength ratio and porosity for SLWFM.

**Figure 17:**
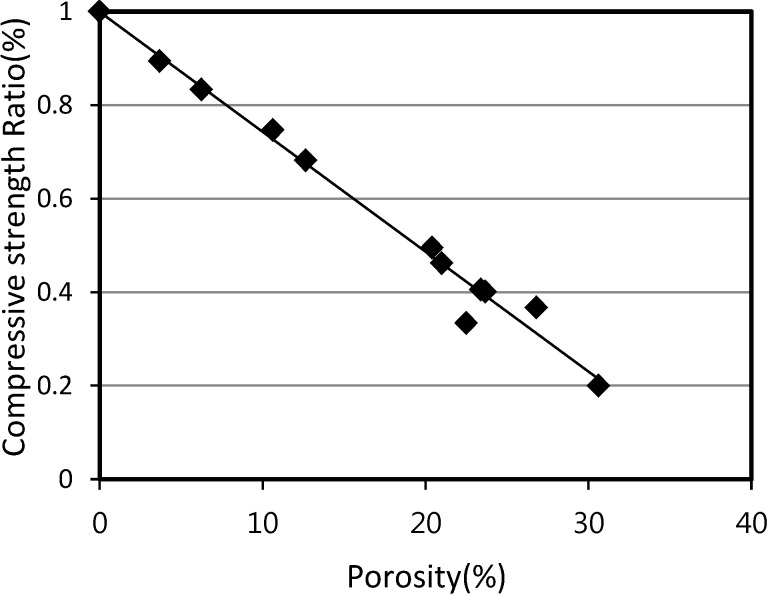
Relationship between compressive strength ratio and porosity for SLWFC.

These compressive strength values at 0% porosity were used to calculate the compressive strength ratio in Figure 16 and Figure 17. Compressive strength ratios for mortar and concrete were calculated by dividing the calculated value of the compressive strength at 28 days by the compressive strength value at porosity of 0% that was calculated earlier.

### 4.2. Estimation of Compressive Strength of Structural Lightweight Foamed Mortar and Concrete

Figure 16 and Figure 17 show the relationship between the compressive strength ratio and the porosity of structural lightweight foamed mortar and concrete. Based on the assumption that the maximum compressive strength ratio is equal to 1 at a porosity of 0%, Figure 16 and Figure 17 can be generated by plotting the calculated compressive strength ratios at different porosities and with different W/B ratios. 

The gradient of the straight line of both mortar and concrete can be used in Equations 6 and 7. Thus, the compressive strength (*F*_am_ and *F*_ac_) of foaming agent dosages for mortar and concrete can be calculated according to:

*F*_am_ = (1 − 0.0226*A*_p_)*F*_m_ (6)

*F*_ac_ = (1 − 0.0253*A*_p_)*F*_c_ (7)

**Figure 18:**
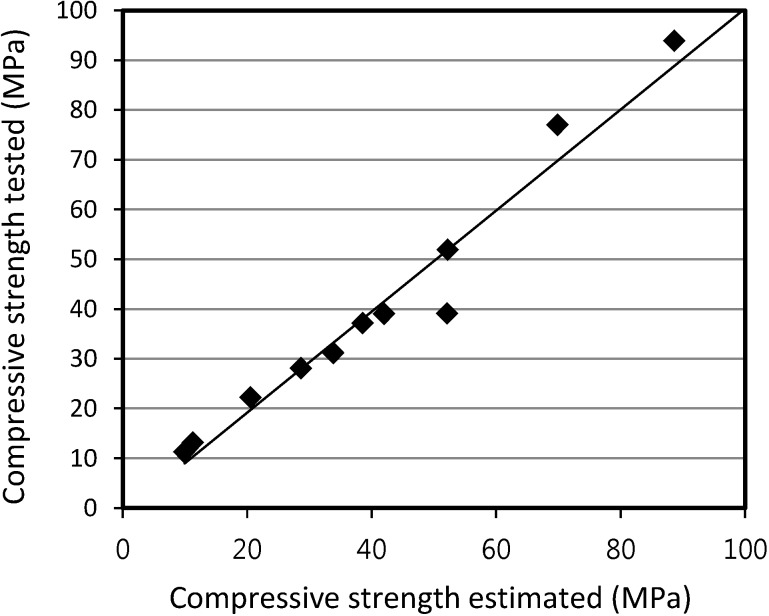
Relationship between tested and estimated compressive strength values for SLWFM.

**Figure 19:**
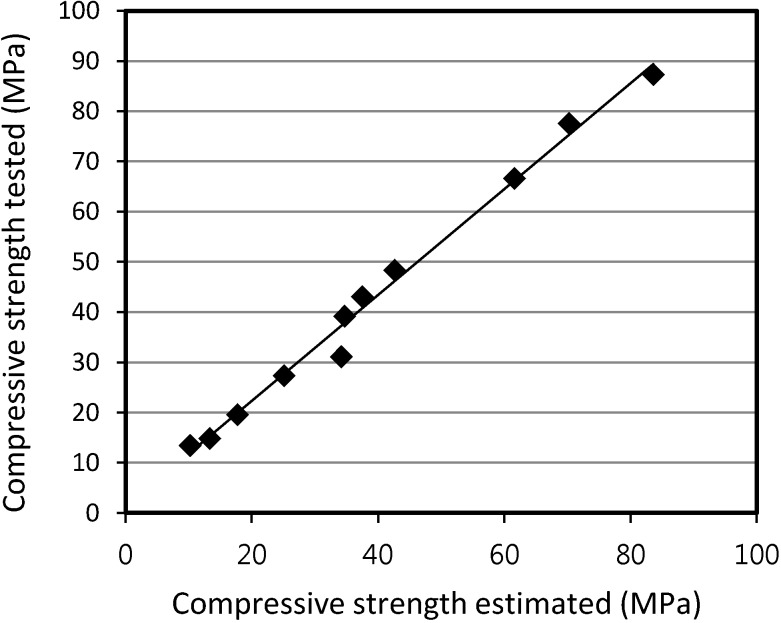
Relationship between tested and estimated compressive strength values for SLWFC.

Figure 18 and Figure 19 show the relationship between the tested and the estimated compressive strength values for SLWFM and SLWFC. The results show that at different W/B ratios, both estimated and tested values were in good agreement and within a 10% error range.

Furthermore, the effects of long term creep, SLWFC pumpability and workability, shrinkage and fire are important factors and are currently addressed in the ongoing study.

## 5. Conclusions

To study the feasibility of developing structural lightweight concrete using normal coarse aggregates and foaming agent admixture, experiments were conducted on specimens to measure and estimate compressive strength of SLWFC. The following conclusions may be drawn:

- The increase in the foaming agent dosage results in the development of pores and, as a result, the density of mortar and concrete decreases.
- In order to obtain higher compressive strength, it is essential that the matrix of the concrete should be improved. Therefore, it is judged that creating pores through foaming agent addition to ultra-high strength concrete would be more efficient to produce structural lightweight concrete.
- In the scope of this study, the maximum dosage of foaming agent to produce independent foams was 0.6% by binder weight and the SEM analysis verified the existence of independent foam formation with sizes of 50–100 μm.
- It is possible to calculate the modulus of elasticity and compressive strength of structural lightweight foamed concrete from the measured values of modules of elasticity and compressive strength of concrete without foaming agent.
- This study confirms that structural lightweight concrete can be made with normal coarse aggregates and foaming agent.
